# Quantifying the reproductive progression of sunflower using FIJI (Image J)

**DOI:** 10.1016/j.mex.2022.101879

**Published:** 2022-10-09

**Authors:** Ana Claudia Ochogavía

**Affiliations:** Instituto de Investigaciones en Ciencias Agrarias de Rosario (IICAR – CONICET – UNR), Campo Experimental ‘J. F. Villarino’, Zavalla, Santa Fe, Argentina

**Keywords:** Bioinformatics, image analysis, flower phenophases, reproductive development, sunflower crop, sunflower reproductive biology, sunflower seed counting, PN, Particles number, DF, Disc flowers number, R, Reproductive stage of the sunflower capitulum, E, Phenophase of the disc flower

## Abstract

Sunflower (*Helianthus annuus* L.) is today the third leading oilseed crop in the world and seed yield is a valuable trait for breeders and researchers. The sunflower capitulum is composed of 700 to 3000 individual flowers on a flattered receptacle. Most reproductive stages (R5 to R6) have at least two disc flower phenophase's coexisting in the same receptacle (E1 to E4). Today, researchers in agroecology and breeders manually quantify the number of disc flowers that achieve the anthesis at different developmental stages of the receptacle. The presented method applies a bioinformatic tool to estimate: (1) the number of disc flowers of each phenophase that are constituting the sunflower´s capitula at different reproductive stages and, (2) the number of developing seeds of each sunflower capitulum. The ImageJ software was used as an image-analysis tool on sunflower capitula photographs. A use case and method validation for each presented protocol is provided. This method will contribute to correlation analysis in agroecological studies and also would be useful for the early prediction of seed yield in breeding programs.

• This is a simple method for the estimation of the number of disc flowers at each phenophase in the sunflower receptacle.
• It is based on integrating the knowledge of sunflower reproductive development with an open-source image analysis platform applied in single workflows.
• This is a precise, non-destructive, rapid, and low-cost method; thus, it has the potential to be adopted as a phenotyping tool for sunflower breeding and research in agroecology.

## Graphical abstract

**Figure:**
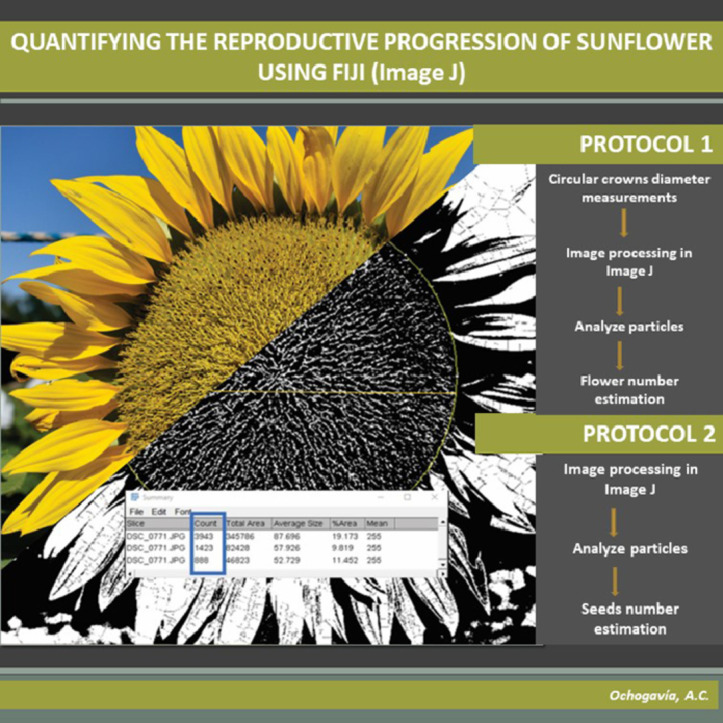

Specifications table

**Table:** 

Subject Area:	Bioinformatics
More specific subject area:	*Image analysis; Reproductive development measurement*
Method name:	*Protocol 1: Estimation of the number of disc flowers of each phenophase at different capitulum reproductive stages* *Protocol 2: Estimation of the number of developing seeds of each sunflower capitulum*
Name and reference of original method:	Abrámoff, M. D., Magalhães, P. J., Ram, S. J. (2004) Image processing with ImageJ. Biophoton Int 11, 36–42. https://10.1002/9780470089941.eta03cs9
Resource availability:	*• Hardware* *- Digital photographic camera (with a minimum resolution of 10 Mpx)* *- Windows-based computer (note that MacOS X and Linux are also supported)* *- Network and Internet connection, with any needed passwords.* *- High-resolution monitor >24in, or dual monitors* *• Software* *- Current version of Fiji (downloaded from*https://imagej.net/Fiji.html#Downloads*). To install ImageJ on a computer with Java pre-installed, or to upgrade to the latest full distribution, download the ZIP archive (6MB) and extract the ImageJ directory.* *-Microsoft Excel or any other spreadsheet program such as Google Docs Spreadsheet or Excel Web Application.*

## Overview

Seed yield in sunflower (*Helianthus annuus* L.) is probably one of the most important traits for crop breeders and researchers. However, some aspects of reproductive biology have received little attention despite its clear linkage with viable seed production. *H. annuus* has a discoid heterogamous capitulum in which the outermost two whorls of flowers are ligulate and sterile. The bisexual disc flowers have a five-lobed corolla with five epipetalous stamens (Miller, 1987) [2]. The cultivated sunflower capitulum is composed of 700 to 3000 individual flowers arising at the same level on a flattered receptacle. The spiral phyllotaxis is the typical pattern of maturation of the successive series of flower buds, with the peripheral flowers the first to open [4]. As a consequence, peripheral flowers are phenologically advanced with respect to the central ones. The most widely used phenological scale in sunflower is based on observations of visible morphological attributes of the whole plant [8]. The Schneiter and Miller [8] scale is divided into either Vegetative (V) or Reproductive (R) stages of plant development. The vegetative stages categorize plant emergence and true leaves development. The reproductive development is divided into nine stages in which, R1 to R5 categorize the inflorescence from flower buds to the anthesis of the first disc flower rows, R5 to R6 describe the reproductive progression considering the proportion of disc flowers in anthesis in the receptacle, and R6 to R9 categorize the most advanced reproductive stages to the seed's physiological maturity [8]. The phenology of disc flowers was first classified by Miller (1987) [2] including four phenophases: flower buds (E1), anthesis with elongated filaments and anther dehiscence (E2), stylar elongation and subsequent receptive stigma (E3), and the post-pollination stage (E4). Additionally, this scale was enhanced and protracted by anatomical and morphological studies reported by Menéndez et al. [7]. Most sunflower reproductive stages have at least two disc-flowers phenophase coexisting in the same receptacle. Today, researchers in agroecology and breeders manually quantify the number of disc flowers that achieve the anthesis at different developmental stages of the receptacle, registering them one by one.

ImageJ is a widely distributed image-analysis software package with an open-source availability to be used without a license (http://rsbweb.nih.gov/ij/) [1]. This software has a simple interface that automates analysis protocols through the use of macros and plugins in which the image analysis of TIFF and JPEG formats could be useful in biological studies.

This work aims to apply the ImageJ software to quantify the number of disc flowers of each phenophase that are constituting the sunflower´s capitulum at different reproductive stages, and also to estimate the seed numbers in the sunflower capitula before harvest. The first protocol (Protocol 1) involves the integration of the FIJI's apps and the sunflower reproductive development knowledge to quantify the number of disc flowers of each phenophase in the different circular crown areas of the receptacle. Flower counting can be achieved at every developmental stage of the sunflower capitulum (from R5.1 to R6, [8]). The second protocol (Protocol 2) involves Fiji's fast image manipulation to estimate the number of seeds that are developing in the sunflower capitulum. The application of Protocol 2 requires the previous validation analysis between flower numbers and seed production for each genotype and field condition. Once characterized, it allows an early estimation of the seed production in future agricultural seasons. . A use case for each protocol illustrates the strength of the program applied to the reproductive development measurement. The novelty of these two bioinformatics tools is based on integrating the knowledge of sunflower reproductive development and an open-source image analysis platform employed in single workflows that address researchers’ and breeders´ needs.

*Method details

## Strategic planning

The two image analysis protocols require a focused and unsaturated image obtained in a correct photographic framing. It is recommended that images of sunflower capitula be taken at the same moment in the day, if possible, during the early morning preventing ligulate flowers from casting shade on the disc flowers. The capitulum surface should be in the focus line and the full circumference of the receptacle should be included in the photographic frame. Moreover, pixel saturation should be avoided since it will generate inaccurate data [6]. The digital camera software has visual indicators to show when an image is oversaturated and, in some cases, the exposure time should be reduced to correct oversaturation. Images could be saved as TIFF or JPEG.

## Protocol 1: Estimation of the number of disc flowers of each phenophase at different capitulum reproductive stages

Protocol 1 is composed of 14 steps and follows a workflow typically found in the 2D particle analysis used in life sciences but considering delimited areas based on the reproductive morphology of the sunflower capitulum (Supplementary file 1). FIJI (Image J) is used as a JPEG image analyzer of a field-taken photograph of a sunflower capitulum. Images should be taken from R5.1 to R6 developmental stage accordingly to Schneiter and Miller [8] scale. The application of this software allows to delimit the circular crown of the receptacle that contains disc flowers of a single phenophase. The external and internal diameters of different circular crown areas are first registered. Next, the total number of disc flowers is quantified in the circular crown areas of the receptacle using the application ‘Analyze particles’ after the image binarization process. The application of this protocol allows us to obtain the flower number of each phenophase in a specific developmental stage of the sunflower capitulum using a single JPEG image that could be taken by most photographic cameras.

1. On the NIH website download ImageJ https://imagej.nih.gov/ij/download.html
2. For Windows installation, click on the highlighted bundled with 32-bit Java software (version ij153). Click Save to save the file in a folder labeled FIJI.app on your desktop. The download will take 30 min approximately.
3. Click Run to the application file, and follow the instructions for the ImageJ setup wizard to install FIJI on your computer.
4. Once the setup wizard is complete, click Finish to launch ImageJ and display the menu and toolbar on your desktop.
5. Open the File menu item, and select Open. Choose the image to be analyzed from your image folder (Fig. 1A). The image will open onto the desktop, outside the software interphase.

**Fig. 1:**
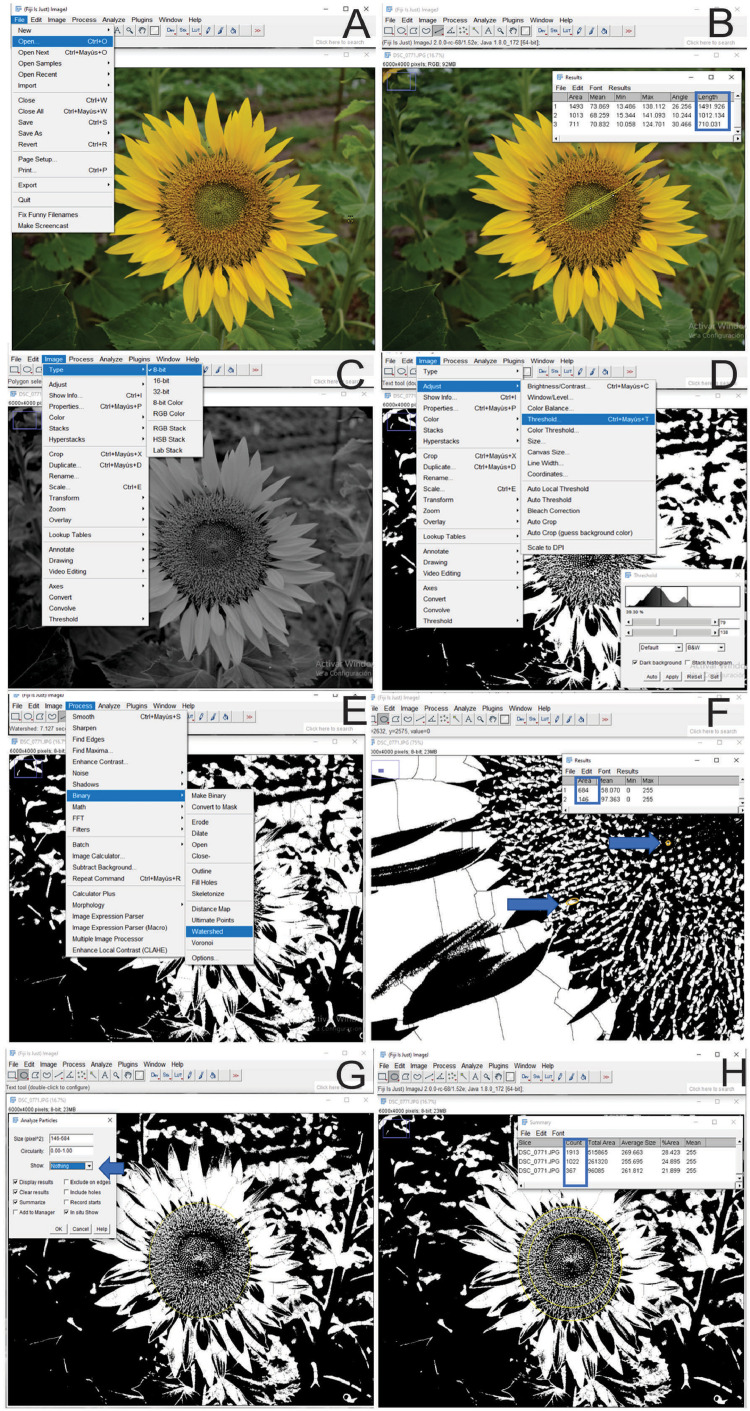
Image analysis workflow of the number of disc flowers estimation using the Analyze Particles tool of the FIJI software. Panel (A) shows the selection of the Image file under the File/Open menu item. Panel (B) describes the capitulum diameter measurement by selecting the line on the menu bar and the option Measure at the Analyze menu item (or Ctrl+M). Panel (C) shows Image 8-bit conversion under the Image /Type 8-bit menu Item. Panel (D) illustrates the location of the Adjust/Threshold and the emerging window to adjust the visual appearance of the capitulum image. Panel (E) describes the image binarization process by selecting Watershed in the Process/Binary menu. Panel (F) shows the measurement of the particle area range, where the smallest and biggest particle area is measured by selecting the oval tool at the menu bar and the option Measure at the Analyze menu item (or Ctrl+M). Panel (G) describes the proper particle counting process in a defined circular area by selecting Analyze Particles from the Analyze menu item and setting the particle area range accordingly to de areas obtained in the previous step (Panel F). Panel (H) shows the final measurement steps where the particle measurement is repeated in all the circumference areas of diameters previously measured (Panel B).
6. Use the Straight-line selection tool at the Barr Menu and extend a line over the capitula circumference diameter. One click is needed to start drawing the line and one click to finish it.
7. Open the Analyze menu item, select Measure (or Ctrl+M) (Fig. 1B), and register the number of pixels on the spreadsheet program. It measures the diameter in pixels of the external circumference of the capitulum. The phenophase of the disc flowers (from E1 to E4, Supplementary file 1) at each circular crown should be registered at this step.
8. Repeat steps 5 and 6 for the inner circular crown areas defined by the different phenophases of the disc flowers (Please see Supplementary file 1) registering the diameter in pixels of each circumference. Flower developmental stages are easily visualized as different textures on the surface of the capitulum.
9. To convert the file to an 8bit image, open the Image menu item and select Type/8-bit (Fig. 1C).
10. Open Image/Threshold (Ctrl+Shift+T) and manually correct the Black and White chromatogram to visualize all the inflorescences (Fig. 1D). Please note that the E3 and E4 phenophases are represented by two spots per flower since the two stigmatic branches are simultaneously visualized at the zenithal imaging (Supplementary file 1).
11. Image binarization is required. Open Process/Binary and select Watershed (Fig. 1E).
12. The particle size can be estimated using the oval selection tool to delimitate the spots defined by a single disc flower, and measure it with Analyze/Measure (Ctrl+M). Particle size should be defined as a range from the area of the smallest particle defined by a disc flower to the biggest particle that could be measured in the receptacle (Fig. 1F). It is necessary to zoom in on the processed image to be able to differentiate the sizes of particles that are generated by each disc flower.
13. Using the Oval selection tool, create the external circle of the longer registered capitulum diameter and select Analyze Particles from the Analyze menu item. Select Show/Nothing, and tick Display Results on the Analyze Particles Menu (Fig. 1G).
14. Repeat Step 12 defining the diameter of the circles according to the sizes registered in step 7 (Fig. 1H).
15. Finally, the number of particles counted from the smallest circle area (diameter Ø 3) is subtracted from the particle number calculated from the middle circle area (diameter Ø 2) to obtain the number of flowers included in the second circular crown area. In the same way, the sum of particles (flowers) from the second circle area (diameter Ø 2) is subtracted from the total of particles of the external circle (diameter Ø 1) to quantify the total of disc flowers of the external circular crown area (Please see Supplementary File 2). This step should be repeated three times to obtain three technical values for each image. The total number of particles counted in a circular crown area of the disc flower at E3 and E4 phenophases should be divided by 2 since each flower is represented by two spots defined by the two stigmatic branches (Supplementary file 1).

## Protocol 2: Estimation of the number of developing seeds of each sunflower capitulum

Protocol 2 is composed of nine steps and, like Protocol 1, is based on the application of FIJI software strength to the knowledge of sunflower reproductive biology. This protocol integrates FIJI (Image J) as a JPEG image analyzer to quantify the total of developing seeds in the sunflower receptacle. Images should be taken at the R6 developmental stage [8] when all the disc flowers achieved the E4 phenophase (Supplementary file 1). The main application used is the ‘Analyze Particles’ that allow to detect the total number of particles in a delimited area. Since genetic, physiological, agronomic, and environmental factors may contribute to seed set and grain fill in sunflower seeds, the application of this protocol requires a previous correlation study between the flower´s number and filled seeds for each genotype and field conditions. Once characterized, it is possible to estimate the number of seeds produced by a single sunflower capitulum before the seeds were formed. applying this protocol to photographs taken on field plants.

The first steps focus on software installation and image visualization. Once adjusted and binarized the image is processed and the following steps are performed for the basic particle analysis of the complete capitulum area. The total of particles counted is the number of seeds produced in a single capitulum.

1. On the NIH website download ImageJ https://imagej.nih.gov/ij/download.html
2. For Windows installation, click on the highlighted bundled with 32-bit Java software (version ij153). Click Save to save the file in a folder labeled FIJI.app on your desktop. The download will take 30 min approximately.
3. Click Run to the application file, and follow the instructions for the ImageJ setup wizard to install FIJI on your computer. Once the setup wizard is complete, click Finish to launch FIJI and display the menu and toolbar on your desktop.
4. Open the File menu item, and select Open. Choose the image to be analyzed from your image folder (Fig. 2A). The image will open onto the desktop, outside the software interphase.

**Fig. 2:**
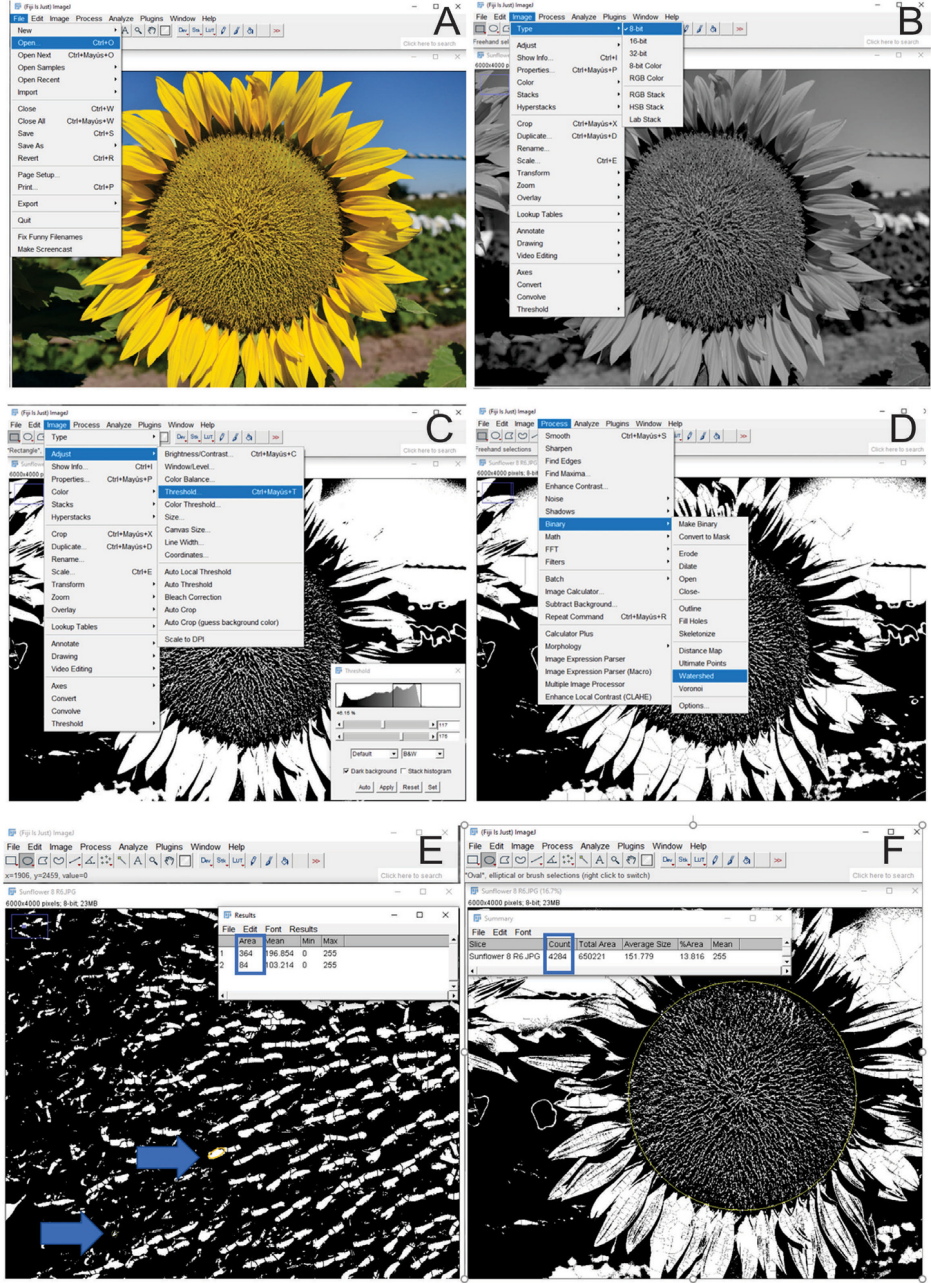
Image analysis workflow of the number estimation of developing seed using the Analyze Particles tool of the FIJI software. As in Fig. 1, Panel (A) shows the selection of the Image file under the File/Open menu item. Panel (B) shows Image 8-bit conversion by Image /Type 8-bit menu Item. Panel (C) illustrates the location of the Adjust/Threshold and the emerging window to adjust the visual appearance of the capitulum image. Panel (D) describes the image binarization process by selecting Watershed in the Process/Binary menu. Panel (E) shows the measurement of the particle area range, where the smallest and biggest particle area is measured by selecting the oval tool at the menu bar and selecting Analyze/Measure (or Ctrl+M). Panel (F) describes the particle counting process in the external capitulum circular area defined by the oval tool at the menu bar, selecting Analyze/Analyze Particles and setting the particle area range accordingly to de areas obtained in the previous step (Panel E).
5. To convert the file to an 8bit image, open the Image menu item and select Type/8-bit (Fig. 2B).
6. Open Image/Threshold (Ctrl+Shift+T) and manually correct the Black and White chromatogram to visualize one white spot in each inflorescence (Fig. 2C).
7. Image binarization is required. Open Process/Binary/Watershed, and adjust manually the threshold (Fig. 2D).
8. The particle size can be estimated using the oval selection tool to delimitate the disc flower area, and measure it with Analyze/Measure (Ctrl+M). Particle size should be defined as a range from the area of the smallest particle defined by a disc flower to the biggest particle that could be measured in the receptacle (Fig. 2E). It is necessary to zoom in on the processed image to be able to differentiate the particle sizes at each disc flower.
9. Using the Oval selection tool, create the external circle of the capitulum diameter and select Analyze Particles from the Analyze menu item (Fig. 2F). The number of particles counted at the E4 phenophase is represented by the number of stigmatic branches detected. Therefore, since each disc flower has two stigmatic flowers, the number of counts should be divided by two to estimate the seed number in the capitulum. This step is repeated three times to obtain three technical values for each image.

An example of the above-described protocol is provided using three sunflower images of the R6 developmental stage (Supplementary sample file), according to the Schneider and Miller [8] phenological scale (Table 2).

## Interpretation of results and method validation

Sunflower is today the third leading oilseed crop in the world, along with soybean and rapeseed [3]. It is the second-most important crop based on hybrid breeding and the fifth-largest oilseed crop in the world [9]. Genetic improvement is a critical factor for this crop productivity optimization and breeding programs require an exhaustive knowledge of reproductive biology. Some sunflower open-pollinated cultivars and wild populations are self-incompatible and are mostly cross-pollinated by insects [2]. With a background of self-incompatibility and seed production dependent on insect vectors, breeding programs have contributed to improving yield over open-pollinated cultivars by producing high levels of self-compatibility in hybrids. Some floral visitors, like pollinators (bees, bumbles, flies, some wasps, etc.), benefit the plant reproduction, but the predators (spiders, some wasps, some beetles, etc.) can damage and interferes the seed production [10]. The insect attraction/repellence is affected by the number of disc flowers achieving the anthesis phenophase E3, where pollen is available for visitors [4], [5],7]. Therefore, the quantification of the number of disc flowers at each phenophase that are constituting the capitula at each reproductive stage will be useful for correlation analysis in agroecological studies that evaluate the interaction of floral visitors and the reproductive development of this crop. Moreover, the estimation of the number of seeds in the capitula of plants at the field would anticipate the seed yield and reduce the time and resources consumed in breeding programs for cultivated sunflower. However, a previous characterization of the seed set and the number of flowers per capitulum should be done for each genotype before applying Protocol 2.

Protocol 1 and 2 allow estimating the number of sunflower disc flowers or seeds at a particular developmental stage in the field cultivated plants. Image acquisition is an important step since the background will vary across the images and will conditionate the particle quantification functions. Therefore, the threshold adjustment is the most important step, one disc flower should be represented by one (E1 and E2 phenophase of the disc flower) or two spots (E3 and E4 phenophase of the disc flower) in the adjusted image. Moreover, since the anthesis of successive series of buds occurs by spiral phyllotaxis, in which peripheral flowers are first to open [4], the most advanced phenophases are at the external circular crown.

An example of the Protocol 1 is provided using six sunflower images at different developmental stages according to the Schneider and Miller phenological scale [8] (Supplementary sample file). The different diameters, the phenophase of the disc flowers at the circular crown area, the total number of particles (PN) quantified, and the number of disc flowers of each phenophase are described in Table 1. The estimation of the number of disc flowers at different phenophases requires the delimitation of each circular crown before the quantification of particles. External diameter (Ø 1), middle diameter (Ø 2), and internal diameter (Ø 3) are first registered in Table 1 (please see Supplementary Material 2) and the different phenophases are registered for each circular crown. After threshold adjustment, image binarization, and particle size range estimation, the circle with de external diameter is defined (Ø 1). Next, the particle number (PN) of the complete circle is counted. This quantification is repeated in circles Ø 2 and Ø 3 (Table 1). To obtain the number of disc flowers of each phenophase, the PN of the internal circles should be subtracted from the external circles. The PN of the central circle is already the total number of disc flowers at E1 phenophase, so, the number of E2 disc flowers is calculated by subtracting the PN of the internal circle from the PN of the middle circle (Ø 2). The E3 disc flowers are calculated by the subtraction of the PN of the middle circle to the external circle (Ø 1), and the resulted PN should be divided by two since each E3 disc flower is defined by two spots at zenithal capitulum images. The total of disc flowers that integrates the capitulum is calculated by the sum of E1, E2, and E3 disc flowers (Table 1). It should be noticed that the capitula from R5.1 to 5.3 developmental stages have disc flowers at E1 and E2 phenophases, in that case, there are only Ø 1 and Ø 2 that define the regions of E2 and E1 disc flowers, respectively.

**Table 1:** Means and standard errors of the estimated disc flower number from images provided in the Supplementary sample file.

Image name	Dev. Stage	Ø 1 (px)	Ø 2 (px)	Ø 3 (px)	PN (Ø 1)	PN (Ø 2)	PN (Ø 3)	Number E3 DF	Number E2 DF	Number E1 DF	Total DF
Sunflower 1 R5.1	R5.1	2291.2	1783.8	NA	1413 ± 6	901.3 ± 7	0	0	511.6 ± 3	901.3 ± 6	1413 ± 8
Sunflower 2 R5.2	R5.2	2193.7	1665.7	NA	1401.3 ± 3	952 ± 7	0	0	449.3 ± 10	952 ± 7	1401.3 ± 8
Sunflower 3 R5.4	R5.4	1884.9	1494.1	1093.6	2133.3 ± 33	1141 ± 11	657.6 ± 4	167.3 ± 10	482.3 ± 9	657.6 ± 2	1308.3 ± 21
Sunflower 4 R5.4	R5.4	1465.8	1062.6	729.5	2065.3 ± 27	753 ± 6	347.6 ± 4	482.3 ± 10	405.3 ± 3	347.6 ± 14	1235.3 ± 15
Sunflower 5 R5.6	R5.6	2328.3	1574.1	960.5	2508 ± 31	625.3 ± 11	327.3 ± 8	777.6 ± 21	298 ± 14	327.3 ± 8	1403 ± 18
Sunflower 6 R5.8	R5.8	2244.1	864.27	568.2	2836.6 ± 37	222.3 ± 7	128 ± 4	1243 ± 18	94.3 ± 10	128 ± 4	1465.5 ± 20

Ø diameter symbol; external diameter (Ø 1), middle diameter (Ø 2), and internal diameter (Ø 3); PN particle number; DF disc flowers; E1, E2, and E3 phenophases of disc flowers.

The second protocol was optimized for the R6 capitula developmental stage, where only one disc flower phenophase is present in the receptacle, E4 phenophase. The receptacle's external diameter (Ø 1) is registered in Table 2. After threshold adjustment, image binarization, and particle size identification, defined the circle with de external diameter and the particle number (PN) of the complete circle are counted. Since in the E4 phenophase the two stigmatic branches are visualized, the total of PN should be divided by two to estimate the number of developing seeds. In some genotypes, in which the receptacle is curved, it is necessary to remove carefully the superior flower organs (corolla, stamens, and style/stigma) to visualize only the ovary. In this case, the PN is just estimating the number of seeds. An example of Protocol 2 is provided using three sunflower images at R6 developmental stages according to the Schneider and Miller phenological scale [8]. The different diameters, the phenophase of the disc flowers at the circular crown area, the total number of particles (PN) quantified, and the number of disc flowers are described in Table 2.

**Table 2:** Means and standard errors of the estimated seed number from images provided in the Supplementary sample file. Variables were calculated from the particles counted into the receptacle circle area.

Image name	Dev. Stage	Ø 1 (px)	PN (Ø 1)	Total of seeds
Sunflower 7 R6	R6	2133.21	3025.6 ± 3.75	1512.83 ± 5.8
Sunflower 8 R6	R6	2469.7	2723.23.3 ± 14	1361.6 ± 7.2
Sunflower 9 R6	R6	2310.1	3633 ± 17.5	1816.5 ± 8.9

Ø diameter symbol; external diameter (Ø 1); PN particle number.

The number of disc flowers of each phenophases were quantified by eye to validate the bioinformatic estimation in the nine capitula analyzed by Protocol 1 and 2. Table 3 shows the total number of disc flowers counted at E1, E2, and E3 phenophases. The number of disc flowers (manually counted) was similar to the number estimated by the above protocols for the nine evaluated capitula (Table 3). Manual counting is a destructive method that requires the dissection of individual flowers and it takes at least one hour per capitulum. On the contrary, both image processing methods are precise, non-destructive, rapid, and low-cost, thus they have the potential to be adopted as phenotyping tools for sunflower breeding and research in agroecology.

**Table 3:** Number of disc flowers (DF) quantify (manually) in the nine capitula that were analyzed by Protocol 1 and 2.

Image name	Dev. Stage	Number E3 DF	Number E2 DF	Number E1 DF	Total DF manually counted	Total DF estimated by Protocol 1 and 2 (Table 1)
Sunflower 1 R5.1	R5.1	0	509	907	1416	1413 ± 8
Sunflower 2 R5.2	R5.2	0	512	916	1428	1401.3 ± 8
Sunflower 3 R5.4	R5.4	163	499	643	1305	1308.3 ±21
Sunflower 4 R5.4	R5.4	497	406	355	1258	1235.3 ±15
Sunflower 5 R5.6	R5.6	805	302	314	1421	1403 ±18
Sunflower 6 R5.8	R5.8	1246	109	135	1490	1465.5 ±20
Sunflower 7 R6	R6	1518	0	7	1525	1512.83 ± 5.8
Sunflower 8 R6	R6	1352	0	2	1354	1361.6 ± 7.2
Sunflower 9 R6	R6	1841	0	0	1841	1816.5 ± 8.9

## Declaration of interests

The authors declare that they have no known competing financial interests or personal relationships that could have appeared to influence the work reported in this paper.

## Data Availability

Data is attached as Supplementary Sample File.

## Appendix Supplementary materials

**Supplementary Figure 1**. Developmental stages of the sunflower capitulum (R5.1; R5.5; R5.7, and R6) and phenological scales of disc flowers (E1 to E4 phenophases). Image, application 1

**Supplementary Sample Files**. List of JPEG images. Sunflower1.JPEG to Sunflower6. JPEGs are provided as a worked-out example for Protocol 1. Sunflower 7.JPEG to Sunflower 9.JPEG are provided as a worked-out example for Protocol 2. Image, application 2
