# Supplementary material for: Quantifying the reproductive progression of sunflower using FIJI (Image J)
Source: MethodsX. 2022 Oct 9;9:101879. doi: 10.1016/j.mex.2022.101879 (PMC9587322; doi:10.1016/j.mex.2022.101879)
Supplement: Supplementary file 1 — Supplementary Figure 1. Developmental stages of the sunflower capitulum (R5.1; R5.5; R5.7, and R6) and phenological scales of disc flowers (E1 to E4 phenophases). [file mmc1.pdf]

**Supplementary Figure 1:** Developmental stages of the sunflower capitulum (R5.1; R5.5; R5.7, and R6) and phenological scales of disc flowers (E1 to E4 phenophases).

The developmental stages are based on the scale described by Schneiter and Miller (1981). At the R5 stage, the anthesis of the tubular flowers initiates at the external region of the receptacle. The ray flowers are fully developed and expanded and all the disc tubular flowers are visible at the receptacle. This stage can be divided into sub-stages depending on the percentage of the capitulum that is in anthesis. For example, if 50% of the capitulum has achieved anthesis, the phenological stage of the capitulum can be considered R5.5. The tubular flowers at different phenological stages are: E1 (flower buds are visualized), E2 (anthesis, anthers emerge and a dark structure can be visualized over the corolla), E3 (two yellow stigmatic branches emerge), E4 (senescence is initiated, the stigmatic branches are dehydrated and curved); and their proportions vary among each Schneiter and Miller's sub-stages.

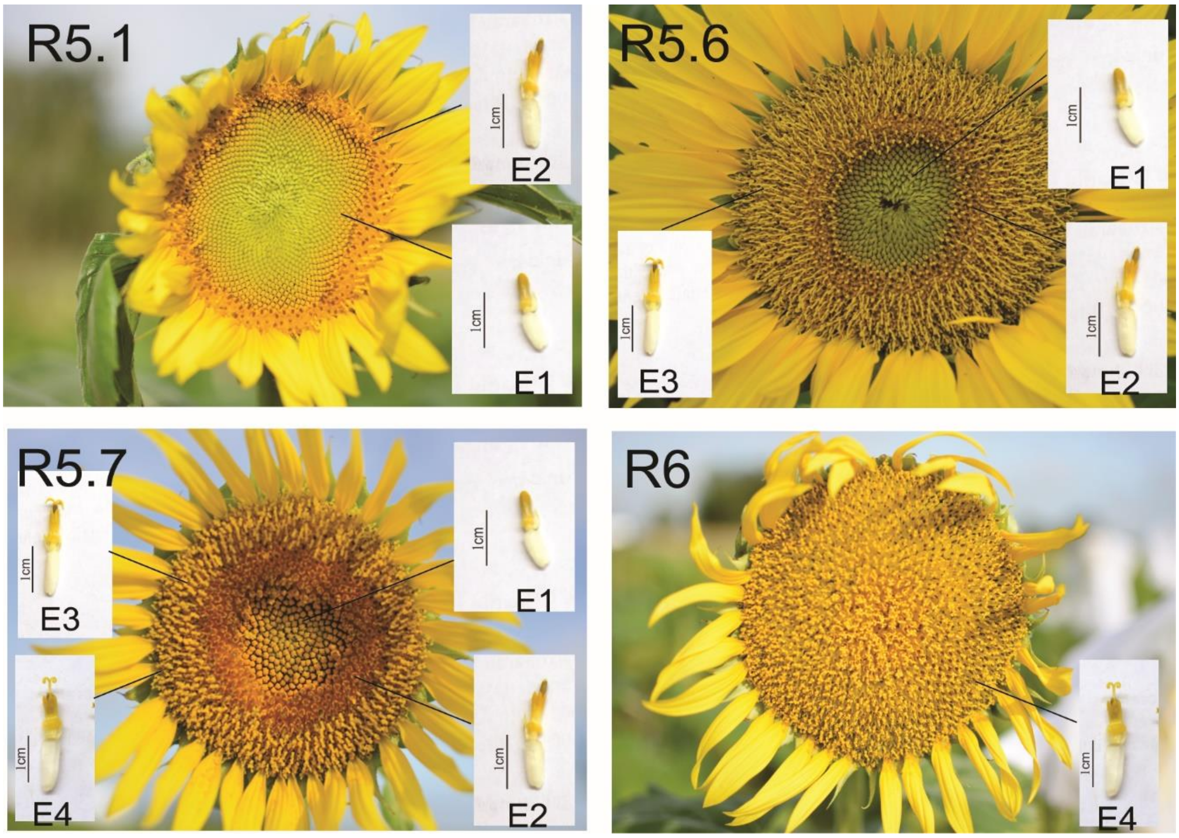


- Schneiter, A.A., Miller, J.F., 1981. Description of sunflower growth stages. Crop Sci 21: 901-903.
